# Minocycline Prevents the Development of Mechanical Allodynia in Mouse Models of Vincristine-Induced Peripheral Neuropathy

**DOI:** 10.3389/fnins.2019.00653

**Published:** 2019-06-27

**Authors:** H. Starobova, A. Mueller, R. Allavena, R. J. Lohman, M. J. Sweet, I. Vetter

**Affiliations:** ^1^Centre for Pain Research, Institute for Molecular Bioscience, The University of Queensland, Saint Lucia, QLD, Australia; ^2^School of Veterinary Science, The University of Queensland, Gatton, QLD, Australia; ^3^School of Pharmacy, The University of Queensland, Woolloongabba, QLD, Australia; ^4^Centre for Inflammation and Disease Research, Institute for Molecular Bioscience, The University of Queensland, Saint Lucia, QLD, Australia

**Keywords:** neuropathic pain, chemotherapy, neuro-inflammation, vincristine, minocycline, toll-like receptor 4, mechanical allodynia, mouse models

## Abstract

Vincristine is an antineoplastic substance that is part of many chemotherapy regimens, used especially for the treatment of a variety of pediatric cancers including leukemias and brain tumors. Unfortunately, many vincristine-treated patients develop peripheral neuropathy, a side effect characterized by sensory, motoric, and autonomic symptoms. The sensory symptoms include pain, in particular hypersensitivity to light touch, as well as loss of sensory discrimination to detect vibration and touch. The symptoms of vincristine-induced neuropathy are only poorly controlled by currently available analgesics and therefore often necessitate dose reductions or even cessation of treatment. The aim of this study was to identify new therapeutic targets for the treatment of vincristine-induced peripheral neuropathy (VIPN) by combining behavioral experiments, histology, and pharmacology after vincristine treatment. Local intraplantar injection of vincristine into the hind paw caused dose- and time-dependent mechanical hypersensitivity that developed into mechanical hyposensitivity at high doses, and lead to a pronounced, dose-dependent infiltration of immune cells at the site of injection. Importantly, administration of minocycline effectively prevented the development of mechanical hypersensitivity and infiltration of immune cells in mouse models of vincristine induce peripheral neuropathy (VIPN) based on intraperitoneal or intraplantar administration of vincristine. Similarly, Toll-like receptor 4 knockout mice showed diminished vincristine-induced mechanical hypersensitivity and immune cell infiltration, while treatment with the anti-inflammatory meloxicam had no effect. These results provide evidence for the involvement of Toll-like receptor 4 in the development of VIPN and suggest that minocycline and/or direct Toll-like receptor 4 antagonists may be an effective preventative treatment for patients receiving vincristine.

## Introduction

Vincristine is part of many chemotherapy regimens and one of the most important antineoplastics for the treatment of a range of pediatric malignancies including medulloblastomas and neuroblastomas. The principle mode of action of vincristine involves binding to β-tubulin and subsequent inhibition of formation of microtubules, an action that interrupts cell division, and leads to growth arrest of cancer cells (Starobova and Vetter, 2017).

Unfortunately, most patients receiving vincristine develop a dose-dependent peripheral neuropathy characterized by sensory, motoric, and autonomic disturbances (Ramchandren et al., 2009; Toopchizadeh et al., 2009; Seretny et al., 2014; Lavoie Smith et al., 2015). Sensory symptoms occur most frequently, typically develop within several weeks of treatment, and commonly manifest as numbness, loss of sensory discrimination, tingling, and pain (Barton et al., 2011). Vincristine-induced motor neuropathy manifests as foot drop, gait abnormalities and cramps (Mora et al., 2016). Despite the wide range of analgesic and neuromodulatory treatments available, none successfully control vincristine-induced pain, leading to further reduction in the quality of life of vincristine-treated patients, as well as dose reduction or cessation of the anticancer therapy (Lavoie Smith et al., 2013). Accordingly, intense research efforts have been directed at elucidating the pathophysiology of vincristine-induced peripheral neuropathy (VIPN), and several putative mechanisms have been proposed. A hallmark of VIPN is degeneration of distal sensory axons, secondary demyelination, and nerve fiber loss (Topp et al., 2000; Boehmerle et al., 2014). Although the molecular mechanisms leading to these changes in axonal function and structure remain unclear, altered microtubule function can impair the anterograde and the retrograde axonal transport, which in turn may lead to Wallerian degeneration, and remodeling of axonal membranes (Topp et al., 2000; Boehmerle et al., 2014). Vincristine is also known to cause dysregulation and structural modifications of neuronal mitochondria, leading to activation of apoptotic pathways, alteration in neuronal excitability, and dysfunction of glial cells (Starobova and Vetter, 2017). In addition, systemic administration of vincristine is associated with an inflammatory response, including the expression of integrins, and enhanced chemotaxis of immune cells (Kiguchi et al., 2009; Old et al., 2014).

We sought to identify new therapeutic targets for the treatment of VIPN by combining behavioral experiments, histological examination and pharmacology, and found that vincristine-induced neuropathy develops predominantly due to neuro-inflammatory interactions involving Toll-like receptor 4 signaling.

## Materials and Methods

### Animals

All behavioral experiments were performed with 8–10 week old adult wild type (C57BL/6J) mice or with *Tlr4*^−/−^ mice of mixed sex, noting that no differences between sexes were observed in preliminary studies (Supplementary Figure S1). Animals were housed with rodent chow and water *ad libitum* in groups of three to five per cage under 12-h light-dark cycles and acclimatized to experiments as described previously (Yin et al., 2016). All experiments were performed in accordance with the *Animal Care and Protection Regulation Qld* (2012), the *Australian Code of Practice for the Care and Use of Animals for Scientific Purposes*, 8th edition (2013) and the *International Association for the Study of Pain Guidelines for the Use of Animals in Research*. Ethical approval was obtained from the University of Queensland animal ethics committee. All behavioral assessments were performed by a blinded observer, unaware of the treatment and genotype of each mouse, with animals randomized to treatment groups.

### Vincristine Administration

**FIGURE 1:**
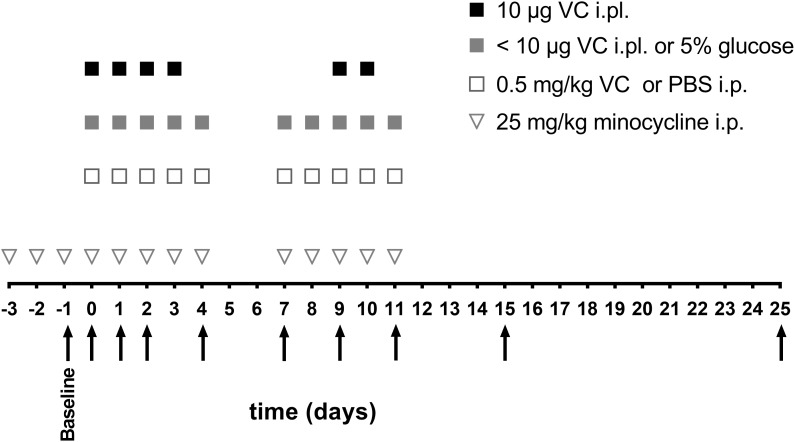
Schedule of vincristine injections and behavioral measurements. Vincristine (<10 μg i.pl. or 0.5 mg/kg i.p.) or vehicle (5% glucose i.pl. or PBS i.p.) (open square, gray square) were administered for five consecutive days, with 2 days break, followed by another five consecutive injections. The highest vincristine dose (10 μg i.pl.) (black square) was administered for four consecutive days, followed by 5 days break and another two injections. Minocycline (25 mg/kg i.p.) (open triangle) was administered once daily, 3 days before vincristine administration, and then with each vincristine injection. Arrows indicate the time points of behavioral assessments, which were always performed before the next vincristine injection with the exception of day 0, where behavioral assessment was performed 3 h after the first vincristine injection.

Vincristine sulfate (Sapphire Bioscience, Australia) was dissolved in sterile Dulbecco’s phosphate buffered saline (PBS) for intraperitoneal (i.p., 0.5 mg/kg, cumulative dose: 5 mg/kg) injection or in sterile-filtrated 5% glucose solution for intraplantar (i.pl.; 1 pg (cumulative dose: 10 pg), 10 pg (cumulative dose: 100 pg), 100 pg (cumulative dose: 1 ng), 1 ng (cumulative dose: 10 ng), 10 ng (cumulative dose: 100 ng), 100 ng (cumulative dose: 1 μg), 1 μg (cumulative dose: 10 μg), and 10 μg (cumulative dose: 60 μg) injection. Vincristine or vehicle solution (5% glucose or PBS) was administered via intraperitoneal injection (10 μl/g) as described previously (Deuis et al., 2013; Old et al., 2014) or via shallow subdermal intraplantar injection into the right hind paw (10 μl) under isoflurane anesthesia (3%) using the injection schedule shown in Figure 1. Intraperitoneal (0.5 mg/kg) and intraplantar (1 pg, 10 pg, 100 pg, 1 ng, 10 ng, 100 ng, and 1 μg) doses were administered once a day for 5 days, followed by a 2 day break and a further five injections; the 10 μg intraplantar dose was administered once a day for 4 days, followed by a 5 day break, and a further two injections.

### Drug Treatment

Minocycline, meloxicam, and gabapentin (Sigma-Aldrich) were dissolved in PBS. Minocycline (25 mg/kg) or PBS were administered by intraperitoneal (i.p.) injection once daily, beginning 3 days prior to the first vincristine administration and then together with each vincristine (i.pl. or i.p.) injection using the injection schedule shown in Figure 1. The dose of minocycline was based on effective doses reported in previous studies of chemotherapy-induced neuropathy (Boyette-Davis and Dougherty, 2011; Boyette-Davis et al., 2011). Meloxicam (5 mg/kg) or gabapentin (100 mg/kg) were administered once by i.p. injection 24 h after a single vincristine injection (100 ng, i.pl.) and behavioral assessment was performed 30 min after meloxicam and gabapentin administration. The doses and timing of meloxicam and gabapentin administration were based on models of burns pain and cisplatin-induced neuropathic pain in which these compounds provide effective analgesia and plasma concentration studies (Deuis et al., 2014; Yin et al., 2016; Pugh et al., 2017; Adrian et al., 2018).

### Mechanical Threshold Measurements

Changes in mechanical paw withdrawal thresholds were assessed using an electronic von Frey apparatus (MouseMet, Topcat Metrology Ltd., United Kingdom) as previously described (Deuis et al., 2017a). After 30 min acclimatization to the MouseMet test enclosures, the ipsilateral (right, for i.pl. and i.p. administration) hind paw withdrawal threshold was determined by measuring the force required to elicit paw withdrawal in response to increasing force applied via a flat-tipped filament. The average of three measurements per mouse was computed as one biological replicate.

### Thermal Threshold Measurements

Thermal threshold measurements were conducted using a MouseMet Thermal (Topcat Metrology Ltd., United Kingdom) device as previously described (Deuis and Vetter, 2016). The thermal probe was preheated to 37°C before applying it to the plantar surface of the ipsilateral (right, for i.pl. and i.p. administration) hind paw. The heat rate was set to 2.5°C/sec, with a cut off set at 55°C to prevent tissue damage and the temperature that elicited paw withdrawal was recorded. The average of three measurements per mouse constituted one replicate.

### Gait Analysis

Gait analysis was performed using the CatwalkXT (Noldus Information Technology, Netherlands) as described previously (Parvathy and Masocha, 2013). The green intensity of the walkway was set at 0.10 and camera gain at 20.00. Only runs of 3–12 s duration with speed variances below 80% were considered for analysis, which was performed using the CatwalkXT software.

### Paw Thickness

The paw thickness of the ipsilateral and contralateral hind paw was assessed using digital Vernier calipers (Kincrome, VIC, Australia), as described previously (Deuis et al., 2017b). Mice were anesthetized using 2% isoflurane and the distal-proximal axis at the metatarsal level of the hind paw was measured 30 min after each vincristine injection.

### Hematoxylin and Eosin (H&E) Staining

C57BL/6J or *Tlr4*^−/−^ mice were injected with vincristine (i.pl., 10 μg, 100 ng or i.p., 0.5 mg/kg) or with vehicle (i.pl., 5% glucose or i.p., PBS) using the injection schedule shown in Figure 1. After 24 h or on day seven, the mice were anesthetized with 10 mg/kg xylazine and 100 mg/kg ketamine i.p. and transcardially perfused with ice-cold PBS followed by ice-cold 4% paraformaldehyde in PBS (Sigma-Aldrich). The injected hind paws were dissected and post fixed for 16 h in 4% paraformaldehyde at 4°C. The paws were incubated at 37°C in decalcification solution containing 20% EDTA and 3% citric acid (both Sigma-Aldrich), pH 7.2 for 14 days followed by 48 h incubation in 50% Organic Compound Tissue (OCT, Tissue – Tek) in PBS. Finally, the paws were frozen in OCT (Tissue-Tek). Tissue was cut with a Leica cryostat CM1950 (Leica Biosystems). The paws were cut into 5 μm thick sections and were stained with hematoxylin and eosin in a Leica ST5020 Autostainer (Leica Biosystems, Mt Waverly, VIC, Australia). Pictures were obtained using the wide-field Zeiss AxioImage M2.m Microscope and Axiocam 506 camera. Three animals per group and 5 sections per animal were analyzed according to the scoring criteria detailed in Supplementary Table S5 by a blinded veterinary pathologist. A representative picture for each group is shown.

### Data and Statistical Analyses

Data and statistical analyses were performed using GraphPad Prism Version 7.00. Statistical significance was determined as ^∗^*P*- adjusted <0.05 and was calculated using two-way ANOVA with Tukey’s multiple comparisons test. All data are shown as mean ± standard error of the mean (SEM); *n* = 6 for all groups (3 females and 3 males), all experimental groups were compared to vehicle receiving group (control). The ED_50_ was calculated using the area under the curve of the experimental values following the i.pl. administration of 1 pg, 10 pg, 100 pg, 1 ng, 10 ng, 100 ng, 1 μg, and 10 μg vincristine.

## Results

### A Novel Mouse Model Replicates Several Symptoms of Vincristine-Induced Peripheral Neuropathy

**FIGURE 2:**
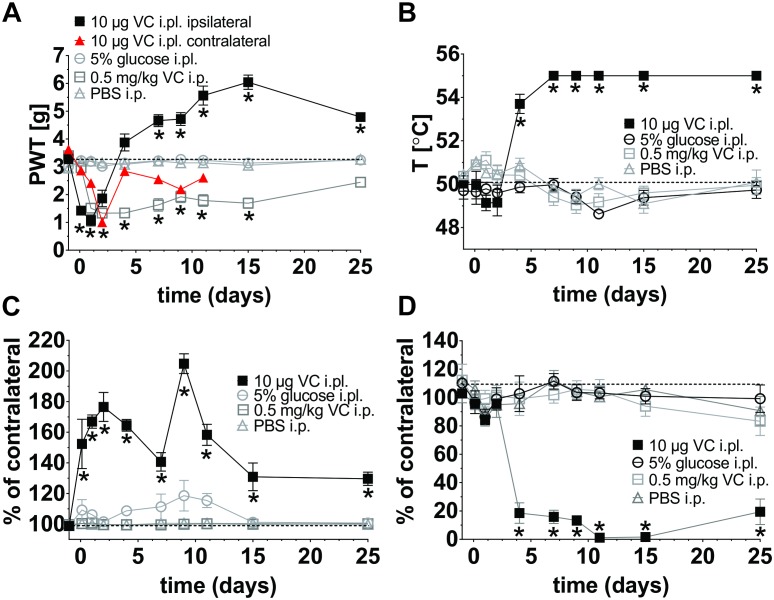
Behavioral characterization of a novel mouse model of vincristine-induced peripheral neuropathy (VIPN). Single daily injection of vincristine (i.pl; 10 μl solution containing 10 μg or i.p.; 10 μl/g solution containing 0.5 mg/kg) or vehicle (i.pl; 10 μl 5% glucose or i.p.; 10 μl/g PBS) were administered using the schedule in Figure 1. Dotted lines indicate baseline values. **(A)** Mechanical paw withdrawal threshold (PWT) following vincristine administration, measured using an electronic von Frey instrument (MouseMet, TopCat Metrology. **(B)** Thermal PWT following vincristine administration assessed using MouseMet Thermal (TopCat Metrology). **(C)** Paw thickness 30 min after injection was assessed using a digital Vernier caliper (Kincrome, VIC, Australia). **(D)** Ipsilateral paw print area expressed in percent of the contralateral paw following vincristine administration was assessed using the CatwalkXT platform (Noldus Information Technology, Netherlands). Print area is the average of paw contact area with the glass platform (in cm^2^). Statistical significance was determined using two-way ANOVA with Tukey’s multiple comparisons test; the experimental groups (vincristine i.pl. or i.p.) were compared to the respective vehicle control group (i.pl., 5% glucose or i.p., PBS). No significant differences between the sexes were detected. All data are shown as mean ± SEM; *n* = 6 for all groups (3 females and 3 males). *^∗^p <*0.05.

Mouse models based on systemic administration of vincristine induce symptoms of mechanical allodynia, but poorly replicate important symptoms of human neuropathy such as sensory loss or gait disturbances. We thus sought to isolate the dose-dependent actions of vincristine on peripheral sensory neurons, we established a mouse model of VIPN based on the local administration of vincristine (1 pg, 10 pg, 100 pg, 1 ng, 10 ng, 100 ng, 1 μg, and 10 μg) via shallow subcutaneous (intraplantar, i.pl.) injections into the hind paw of C57BL/6J mice (Figure 1). We then compared the resulting phenotypes to that of a conventional mouse model of VIPN based on systemic intraperitoneal (i.p.) administration. Local administration of vincristine caused a dose- and time-dependent mechanical hypersensitivity that developed slowly at lower doses of vincristine (≤10 ng) and rapidly at higher doses (≥100 ng), with a calculated ED_50_ of 3.7 ng (Figure 2A, Supplementary Figure S2, and Supplementary Table S1). Intraplantar injection of vehicle (5% glucose) did not affect mechanical thresholds. Interestingly, besides causing initial mechanical hypersensitivity, local administration of higher vincristine doses (10 μg and 1 μg i.pl.) also led to the development of a more slowly developing mechanical hypoalgesia, as evidenced by an increase in the mechanical paw withdrawal threshold above baseline (Figure 2A, Supplementary Figure S2, and Supplementary Table S1). This effect occurred with a calculated ED_50_ of 924.5 ng, and is consistent with the clinical symptomatology, which includes hypoesthesias that typically develop at higher cumulative vincristine doses (Lieber et al., 2018). An apparent recovery from the hypoalgesia, evidenced by a return of the mechanical paw withdrawal thresholds toward baseline values (dotted line) was observed after 25 days (Figure 2A, Supplementary Figure S2, and Supplementary Table S1).

For comparison, we additionally assessed a conventional mouse model based on multiple intraperitoneal (i.p.) injections of vincristine (0.5 mg/kg; Figure 1). As previously reported, in this model a significant decrease of the mechanical threshold, beginning on day 2 and persisting for at least 2 weeks, was observed (Figure 2A, Supplementary Figure S2, and Supplementary Table S1). Although vincristine is not administered by subcutaneous injection clinically, this route achieves high local concentrations at peripheral nerve endings, and has provided considerable insight into the pathophysiological mechanisms of other chemotherapy-induced neuropathies (Deuis et al., 2013, 2014). Interestingly, unilateral intraplantar administration of vincristine, at the highest dose tested (i.pl., 10 μg), also elicited a more slowly developing mechanical allodynia in the contralateral paw (Figure 2A and Supplementary Figure S1), consistent with s.c. administration of vincristine eliciting systemic effects similar to i.p. administration. Similar contralateral effects were also observed with 1 μg (i.pl.) vincristine, but not with 100 ng (i.pl.) or lower doses (data not shown). No changes of the thermal threshold, paw swelling, or gait parameters of the contralateral hind paw were observed (data not shown).

Vincristine-treated patients regularly develop a loss of sensory discrimination including difficulties to discriminate between hot and cold temperatures (Barton et al., 2011). We therefore assessed if our model replicates this symptom and found that administration of vincristine indeed caused dose-dependent thermal hypoalgesia that was only apparent at higher vincristine doses (10 μg and 1 μg i.pl.; Figure 2B, Supplementary Figure S2, and Supplementary Table S2), while administration of the lower dose (100 ng i.pl.) caused a small increase in thermal threshold that was only significant at day 9. Only minor, non-significant decreases in heat thresholds were observed at vincristine doses <10 ng (Supplementary Figure S2, and Supplementary Table S2), and thus overall, vincristine caused thermal hypoalgesia with an ED_50_ of 470 ng based on the area under the curve over baseline for each vincristine dose. The intraperitoneal administration of vincristine caused a small, non-significant decrease in thermal threshold on day 7, 9, and 11 compared with vehicle (Figure 2B, Supplementary Figure S2, and Supplementary Table S2).

A striking dose-dependent inflammatory response, evidenced by local redness and swelling of the injected paw, was visible within 30 min after intraplantar injection of doses as low as 1 ng, but not after i.pl. injection of vehicle (5% glucose) (Figure 2C, Supplementary Figure S2, and Supplementary Table S3). The intraperitoneal injection of vincristine or PBS did not cause any changes of paw thickness at any measured time point (Figure 2C and Supplementary Table S3).

Vincristine-induced motor neuropathy manifests in patients as a foot drop, gait abnormalities and cramps (Mora et al., 2016). We thus performed gait analysis using the CatwalkXT platform (Noldus Information Technology, Netherlands). The weight bearing parameter (print area) of animals receiving the highest dose (i.pl., 10 μg) of vincristine showed significant (^∗^*p* < 0.05) differences when compared to vehicle-treated (i.pl., 5% glucose) animals from day 4, with slow apparent recovery on day 25 (Figure 2D and Supplementary Table S4). A significant, though less pronounced, change of print area (day 7, 9, 11, and 25) was observed with 1 μg vincristine (i.pl.), while neither 100 ng (i.pl.) nor glucose (i.pl.) caused significant gait changes. The intraperitoneal injection of vincristine or PBS did not cause any changes in weight bearing (Figure 2D and Supplementary Table S4).

### Histopathological Evaluation Reveals Local Infiltration of Leukocytes After Vincristine Treatment

**FIGURE 3:**
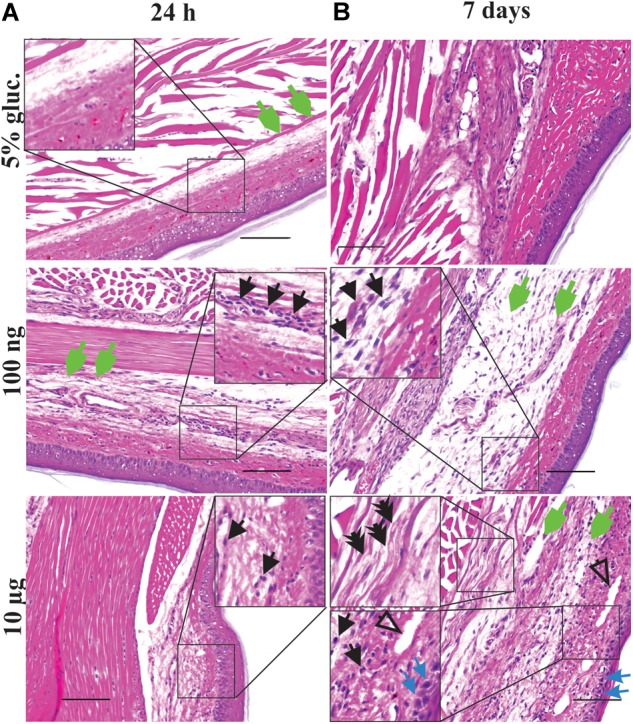
Local vincristine administration leads to infiltration of leukocytes. Representative pictures (H&E staining) showing histopathological changes of the epidermis, dermis, hypodermis and adjacent areas of the plantar glabrous skin of the injected hind paw after administration of vincristine (i.pl.,10 μg or 100 ng vincristine) or vehicle (i.pl., 5% glucose) after 24 h **(A)** or 7 days **(B)**. Injections were performed as indicated in Figure 1. Magnification 200 x; scale bar (100 μm). Black arrow, leukocytes; open arrow, arteriolar necrosis; green arrow, edema; blue arrow, dermal necrosis; double arrow, nerve vacuolization.

In light of the apparent inflammatory effects of vincristine, we next sought to examine the histopathological changes after vincristine administration using hematoxylin and eosin (H&E) staining. Controls receiving intraplantar 5% glucose injections had minimal morphologic changes. Low-dose of vincristine (i.pl., 100 ng) caused mild muscular and subcutaneous edema and a mild neutrophilic perivascular infiltrate at 24 h although the nerve, bone, skin, and vasculature were morphologically normal (Figure 3A and Supplementary Table S5). These changes remained similar at 7 days, with moderate subcutaneous and muscular edema, some subjective nerve vacuolation, a mild leukocytic infiltrate and minimal fibroblast reaction apparent (Figure 3B and Supplementary Table S5).

The most severe morphological changes in the paw tissue were noted in animals treated with the highest dose (i.pl., 10 μg) of vincristine (Figure 3A,B and Supplementary Table S5), consistent with the vesicant effects of vincristine observed after extravasation (Figure 2C; Ener et al., 2004). At 24 h post-injection, we observed minimal histopathological changes, including mild muscular and subcutaneous edema, some minor nerve vacuolization in a focal area of peripheral nerve and a mild perivascular leukocytic infiltration (likely neutrophils) (Figure 3A and Supplementary Table S5). The morphology worsened at day 7 to regionally extensive, full thickness epidermal necrosis with marked edema of the underlying soft tissues (dermal collagen, muscle, and nerve bundles). The dermis contained a moderate infiltrate of leukocytes, including macrophages and neutrophils as well as rare lymphocytes (Figure 2C). Furthermore, there was expansion of the peripheral nerve bundles in the paw pad by clear fluid (edema) and a finely feathered vacuolization of the myelin sheaths (Figure 3B and Supplementary Table S5).

The systemic delivery of vincristine caused, at 24 h and 7 days, only minimal morphologic changes related to tissue processing (Supplementary Table S5). Sciatic nerves and DRGs examination did not show any visible morphological changes at any dose (data not shown).

### Minocycline Prevents Vincristine-Induced Mechanical Allodynia and Hind Paw Inflammation

In light of the inflammatory changes induced by vincristine, we next sought to elucidate whether these processes contribute to the symptoms of VIPN. Neuro-immune mechanisms have previously been implicated in the development of paclitaxel-induced neuropathy, which was prevented by treatment with minocycline (Boyette-Davis et al., 2011). We thus sought to assess the effects of minocycline on the development of vincristine-induced mechanical allodynia and inflammation using both models of VIPN.

**FIGURE 4:**
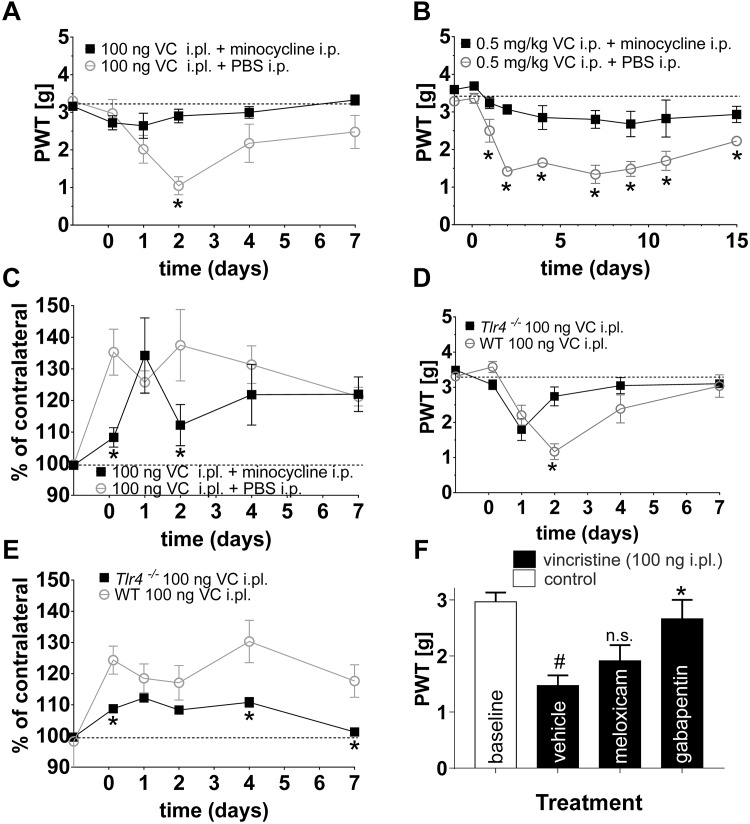
Minocycline prevents the development of vincristine-induced mechanical allodynia in multiple mouse models. **(A,B)** Minocycline (i.p., 25 mg/kg) prevented the development of mechanical hypersensitivity following local [i.pl., 100 ng; **(A)**] and systemic [i.p., 0.5 mg/kg; **(B)**] administration of vincristine (PWT: paw withdrawal threshold). **(C)** Treatment with minocycline (i.p., 25 mg/kg) had a small but significant (^∗^*p* < 0.05) effect on paw swelling following administration of vincristine (100 ng, i.pl.). **(D)** The vincristine-induced decrease in paw withdrawal threshold (i.pl., 100 ng) was significantly increased (^∗^*p* < 0.05 compared with wild-type controls) in *Tlr4*^-/-^ animals at day 2. **(E)** Vincristine-induced paw swelling (i.pl., 100 ng) was significantly decreased (^∗^*p* < 0.05 compared with wild-type controls) in *Tlr4*^-/-^ animals at 3 h and day 4. Dotted line represents baseline values. Minocycline was administered as described in Figure 1. **(F)** Treatment with gabapentin (100 mg/kg i.p.) but not the anti-inflammatory meloxicam (5 mg/kg i.p.) reversed mechanical allodynia elicited by local administration of vincristine (100 ng, i.pl.) ^#^*p* < 0.05 compared with baseline; n.s., *p* > 0.05 compared with vehicle. All data are shown as mean ± SEM; *n* = 6 for all groups (3 females and 3 males) (^∗^*p* < 0.05 compared with vehicle). Statistical significance was determined using two-way ANOVA with Tukey’s multiple comparisons test.

Co-treatment with minocycline (i.p., 25 mg/kg) significantly diminished mechanical hypersensitivity from day 2 of local and from day 1 of systemic vincristine treatment (Figure 4A,B). Similarly, paw swelling following i.pl. administration of vincristine (100 ng) was significantly decreased at 3 h and on day 2 (Figure 4C). There was no significant effect of minocycline treatment on changes in thermal threshold (data not shown).

Minocycline has previously been suggested to exert its anti-inflammatory actions via modulation of TLR4 expression (Nazemi et al., 2015). Thus, to further confirm whether TLR4 signaling contributes to the development of mechanical hypersensitivity and paw swelling following local vincristine administration, we also assessed the development of VIPN in *Tlr4*^−/−^ mice. Similar to treatment with minocycline, paw withdrawal thresholds were significantly increased (^∗^*p* < 0.05) in *Tlr4*^−/−^ mice on day two of local vincristine treatment (Figure 4D) compared to wild type (C57BL/6J) animals. Similarly, paw swelling was significantly decreased (^∗^*p* < 0.05) in *Tlr4*^−/−^ mice at 3 h and 4 days of local vincristine treatment (Figure 4E). In contrast, treatment with the anti-inflammatory meloxicam (5 mg/kg i.p.) had no effect on vincristine-induced neuropathy, while the analgesic gabapentin reversed pain behaviors at high dose (100 mg/kg i.p.) (Figure 4F). These data support a TLR4-mediated inflammatory process after both local and systemic vincristine administration resulting in development of neuropathic pain.

**FIGURE 5:**
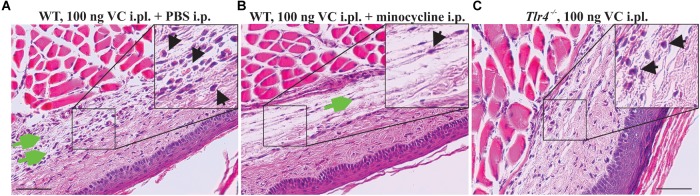
Minocycline prevents infiltration of leukocytes following local administration of vincristine. Representative pictures (H&E staining) showing histopathological changes of the epidermis, dermis, hypodermis, and adjacent areas of the plantar glabrous skin of the injected hind paw after administration of vincristine (i.pl., 100 ng vincristine) and PBS **(A)** or co-treatment with minocycline (i.p., 25 mg/kg) **(B)** in C57BL/6J mice or *Tlr4*^−/−^ mice **(C)** treated with vincristine only (i.pl., 100 ng) after 7 days. Injections were performed as indicated in Figure 1. Magnification 200×; scale bar (100 μm). Black arrow, leukocytes; green arrow, edema.

Consistent with the reduced paw swelling of animals treated with vincristine (i.pl., 100 ng) and co-treated with minocycline (i.p., 25 mg/kg) (Figure 4C), we observed a reduced perivascular infiltrate and reduced fibroblast reaction after 7 days of treatment when compared to control group receiving vincristine (i.pl. 100 ng) and PBS (Figure 5A,B). Additionally, we observed minimal reduction in subcutaneous and muscular edema with absence of epidermal and vascular necrosis. Similarly, *Tlr4*^−/−^ animals treated with vincristine (i.pl., 100 ng) for 7 days showed reduced leukocytic perivascular infiltrate with minimal subcutaneous edema (Figure 5C) when compared to control group receiving vincristine (C57BL6/J, i.pl., 100 ng).

## Discussion

Since the approval of Oncovin by the US FDA in 1963, vincristine has been one of the most important antineoplastics for the treatment of several common cancer types, including a range of brain tumors. However, like many antineoplastics, vincristine causes severe side effects in many patients, of which peripheral neuropathy often drastically reduce quality of life (Sands et al., 2017). In addition, the progressive peripheral sensorimotor neuropathy caused by vincristine can be treatment limiting and presents a challenge to clinicians as dose reductions or cessation of therapy are the only available effective management options (Postma et al., 1993; Mora et al., 2016). Thus, a better understanding of the pathophysiological mechanisms involved in the development of VIPN is urgently needed.

Vincristine is known to exert direct effects on sensory neurons, with axonal degeneration preceding pathological changes at the cell body (Ravula et al., 2007). Accordingly, to delineate the peripheral mechanisms of VIPN, we assessed the pathophysiological effects induced by local intraplantar injection of vincristine. This approach has already provided significant insight into the mechanisms underlying oxaliplatin- and cisplatin-induced neuropathy (Deuis et al., 2013, 2014), and we thus reasoned that a combination of behavioral experiments with histological examination after vincristine treatment would enable the identification of novel therapeutic targets for treatment of VIPN.

Notably, in addition to mechanical hypersensitivity, which also occurs in conventional models of VIPN based on intraperitoneal injection of vincristine, local vincristine administration elicited several symptoms that are common in human patients, including hypoesthesia to both mechanical and heat stimuli as well as gait abnormalities, and signs of inflammation. Similar to humans, the development of these symptoms was dependent on the cumulative dose of vincristine, and was partially reversible. Thus, i.pl. administration of vincristine may be a more suitable model to assess the efficacy of novel therapies and management strategies aimed at improving positive as well as negative symptoms of VIPN. Importantly, the mechanisms leading to VIPN appear to be conserved across the different routes of administration, with both i.p and i.pl. administration leading to mechanical allodynia that is caused by an inflammatory neuropathy.

The effective concentration achieved in tissue, specifically at peripheral nerve terminals, after systemic dosing is unknown. However, the observation that the contralateral paw develops mechanical allodynia after i.pl. injection of the highest doses suggests that the spectrum of symptoms observed in this model is simply a reflection of higher local concentrations, rather than mechanistic differences in pathophysiology. Additionally, the effects seen on the contralateral side may be due to central sensitization of neurons in the spinal cord leading to neuronal hyper-excitability affecting the contralateral paw. Accordingly, allodynia, hypoesthesia, and gait disturbances appear to represent a continuum of pharmacological effects that cannot be achieved through systemic dosing without serious adverse effects. Thus, although i.pl., s.c. or i.p. injection are not routes of administration used in patients, experimental models based on these nonetheless provide important insights into the pathophysiological mechanisms of chemotherapy-induced neuropathy. In the case of vincristine, peripheral extravasation causes vesicant effects, including pain, local swelling, and ulceration in severe cases (Sauerland et al., 2006). Treatment options for extravasation of vincristine include flushing of the injection site as well as treatment with hyaluronidase, which is intended to facilitate dispersal of the drug (Bertelli et al., 1994). Intriguingly, our results suggest that the mechanisms leading to the vesicant effects of vincristine after local administration overlap with the mechanisms leading to VIPN, albeit extravasation leads to extremely high local drug concentrations.

Vincristine binds to β-tubulin (Lobert et al., 1996) and disrupts the function of microtubules, which are not only important during mitosis but also a critical component of neuronal axons. Accordingly, it has been proposed that altered axonal transport causes the progressive sensory-motor neuropathy observed in vincristine-treated patients. However, not all vinca alkaloids cause the same degree of neuropathy, suggesting that alternate pathways may contribute to the pathophysiology of VIPN. Indeed, several studies have implicated inflammatory processes, including the infiltration of macrophages and monocytes to sciatic nerve and dorsal root ganglions (Kiguchi et al., 2009; Old et al., 2014), and release of the cytokines TNF and IL6 (Wang et al., 2012) in the pathophysiology of VIPN.

Consistent with these studies, we observed striking infiltration of immune cells, in particular leukocytes, 24 h after vincristine injection. The precise mechanisms leading to activation of the innate immune system by vincristine remain unclear. One plausible explanation is that cytotoxic effects of vincristine lead to the release of cellular contents, such as ATP, which in turn causes activation of immune cells. Alternatively, vincristine may directly act on immune cells through yet unknown mechanisms. Indeed, the related vinca alkaloid vinorelbine induces production of several cytokines, including IL1ß, IL-6, and IL-12, in cultured dendritic cells and augments stimulation of T-cells without causing overt cell death (Tanaka et al., 2009).

Interestingly, we saw few histopathological neuronal changes apart from an expansion of the peripheral nerve bundle, and finely feathered vacuolization of the myelin sheaths at the highest (i.pl., 10 μg) vincristine dose. In contrast, mechanical hyperalgesia and immune cell infiltration occurred at considerably lower doses and developed comparatively rapidly, suggesting that positive symptoms of VIPN arise from altered neuronal excitability, possibly due to the well-known action of pro-inflammatory cytokines on sensory neurons (Junger and Sorkin, 2000; Binshtok et al., 2008). For example, exposure of DRG neurons to TNF *in vitro* and *in vivo* increases excitability and produces ectopic firing, while IL-1β acts directly on sensory neurons and enhances excitability of nociceptors through modulation of voltage-gated sodium currents (Binshtok et al., 2008). Additionally, vincristine has been shown to induce release of IL-6 in peripheral macrophages (Kiguchi et al., 2008b) and TNF by microglia and astrocytes in the spinal cord of vincristine-treated mice (Kiguchi et al., 2008a). Additionally, chemotherapy agents were shown to have profound inflammatory effects on keratinocytes possibly leading to release of inflammatory mediators (Johnson et al., 1987). An immune-driven pathology is further supported by the anti-allodynic effects observed in Tlr4^−/−^ animals, and after treatment with minocycline. Additionally, the use of TLR4 receptor inhibitors, such as TAK-242, provides further possibilities for the treatment of VIPN, however, those effects should be investigated in future experimental and clinical studies.

Interestingly, neuro-immune mechanisms have previously been implicated in the development of paclitaxel-induced neuropathy and intra-epidermal nerve fiber loss, which was prevented by treatment with minocycline (Boyette-Davis et al., 2011). Minocycline is commonly used as a prototypical inhibitor of peripheral immune cells and microglia (Kobayashi et al., 2013) and has been shown to inhibit the production of proinflammatory molecules in monocytes and microglia (Nikodemova et al., 2007; Pang et al., 2012). Consistent with the inflammatory signature revealed by histological examination after treatment with vincristine, minocycline significantly reduced paw swelling, mechanical hypersensitivity, and minimized leukocytic perivascular infiltration. It is currently unclear whether treatment with minocycline could reverse signs of VIPN that have already developed. However, as chemotherapy treatment is a planned event, anti-inflammatory pre-treatment would be possible in clinical practice. While the anti-inflammatory and anti-apoptotic targets of minocycline remain unknown, this antibiotic has been shown to modulate the activation of microglia and immune cells as well as the release of cytokines, chemokines, and nitric oxide (Sun et al., 2015; Amini-Khoei et al., 2018; Verma et al., 2018). In addition, minocycline decreased the expression levels of Tlr4 in microglia in a model of chronic constriction injury, and several studies have reported that minocycline-induced effects are replicated by inhibition or knockdown of the *Tlr4* gene (Nazemi et al., 2015; Yan et al., 2015). Involvement of Tlr4 signaling pathways was also implicated in the pathophysiology of vincristine-induced neuropathy based on transcriptomic studies assessing gene expression changes in dorsal root ganglion neurons (GEO ref. GSE125003). Consistent with these data, hind paw sensitivity, paw swelling and leukocytic perivascular infiltrate were also reversed in *Tlr4*^−/−^ animals, suggesting that minocycline acts on a common signaling pathway. In contrast, the simple anti-inflammatory cyclo-oxygenase inhibitor meloxicam was ineffective at reversing vincristine-induced inflammation and neuropathic symptoms, confirming that neuro-inflammatory mechanisms underlying VIPN differ from other types of inflammatory pain [e.g., burns-induced pain (Yin et al., 2016)] that are effectively treated by meloxicam.

In a previous study reporting lack of efficacy of minocycline using a rat model of VIPN, the drug was administered by the intrathecal route (Ji et al., 2013). In light of the significant peripheral inflammation we observed, which was paralleled by development of neuropathic symptoms, these data suggest that peripheral inflammatory mechanisms are key drivers of VIPN.

As minocycline is a readily available approved drug with a well-defined adverse event profile, it may be suitable to treat some of the symptoms of VIPN, including pain and swelling arising from local extravasation, and could provide immediate benefit to patients. Moreover, further investigation into the contribution of the TLR4 signaling pathway to the development of mechanical hyperalgesia may provide an opportunity for the development of novel analgesics.

In summary, we demonstrated that local injection of vincristine causes swelling and redness of the injected paw as well as a strong dose-dependent infiltration of immune cells to the site of injection. Consistent with the neuro-inflammatory signature, mechanical allodynia was decreased in mice lacking *Tlr4*, as well as in mice treated with minocycline. Our data thus provide evidence that the innate immune system contributes to the development of VIPN and suggests that Tlr4 may be a novel target for the treatment of vincristine-induced painful hyperesthesia.

## Ethics Statement

This study was carried out in accordance with the Animal Care and Protection Regulation Qld (2012), the Australian Code of Practice for the Care and Use of Animals for Scientific Purposes, 8th edition (2013) and the International Association for the Study of Pain Guidelines for the Use of Animals in Research. Ethical approval was obtained from the University of Queensland animal ethics committee. All behavioral assessments were performed by a blinded observer, unaware of the treatment and genotype of each mouse, with animals randomized to treatment groups.

## Author Contributions

HS, RL, RA, and IV participated in the research design. HS, AM, and RA conducted the experiments. MS contributed with the analytical tools. HS carried out the data analysis. All authors wrote the manuscript.

## Conflict of Interest Statement

The authors declare that the research was conducted in the absence of any commercial or financial relationships that could be construed as a potential conflict of interest.
