# Supplementary figures and images for: Minocycline Prevents the Development of Mechanical Allodynia in Mouse Models of Vincristine-Induced Peripheral Neuropathy
Source: Front Neurosci. 2019 Jun 27;13:653. doi: 10.3389/fnins.2019.00653 (PMC6610325; doi:10.3389/fnins.2019.00653)

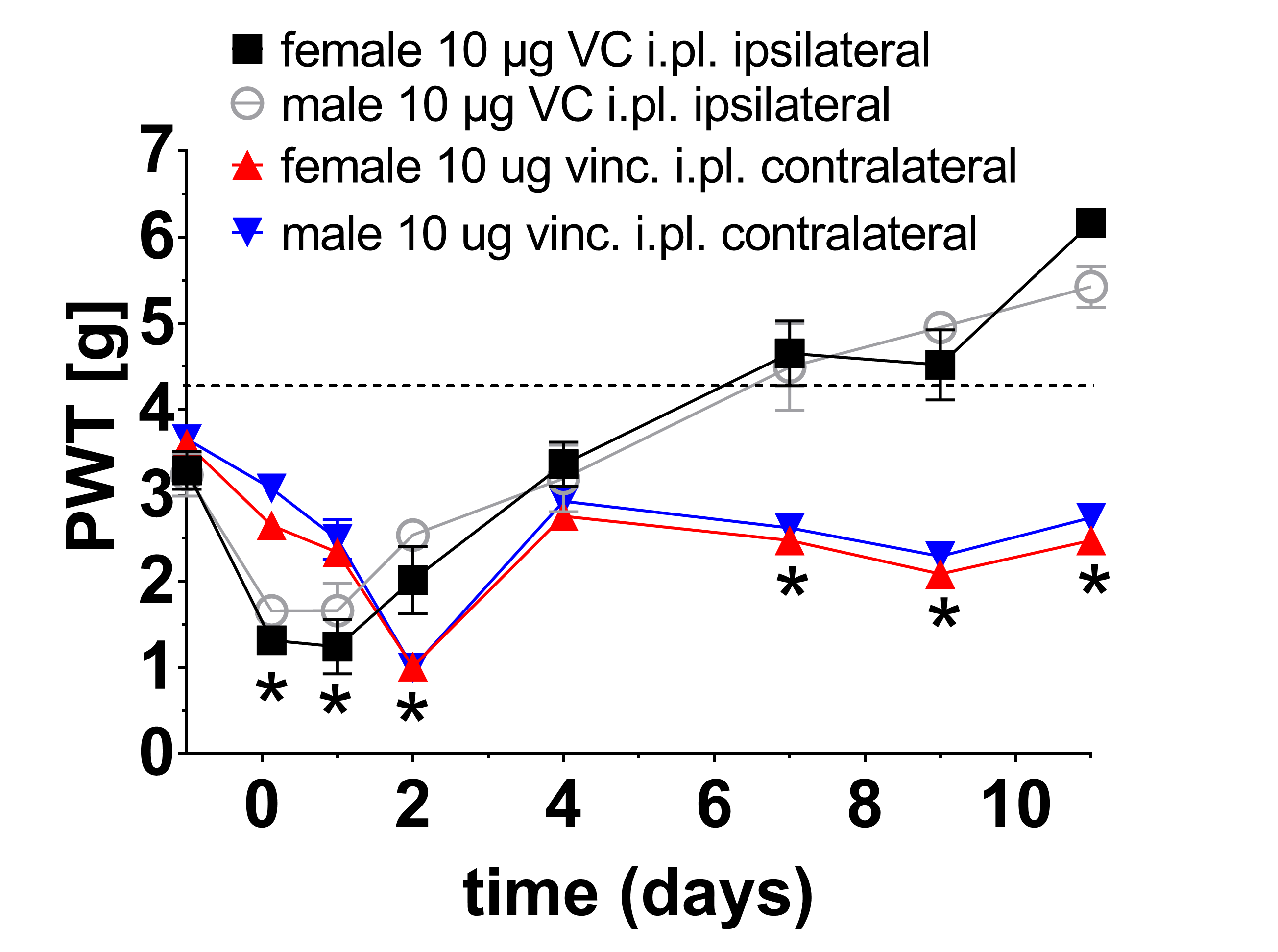

Supplement: Supplementary file 1 [file Image_1.TIF]

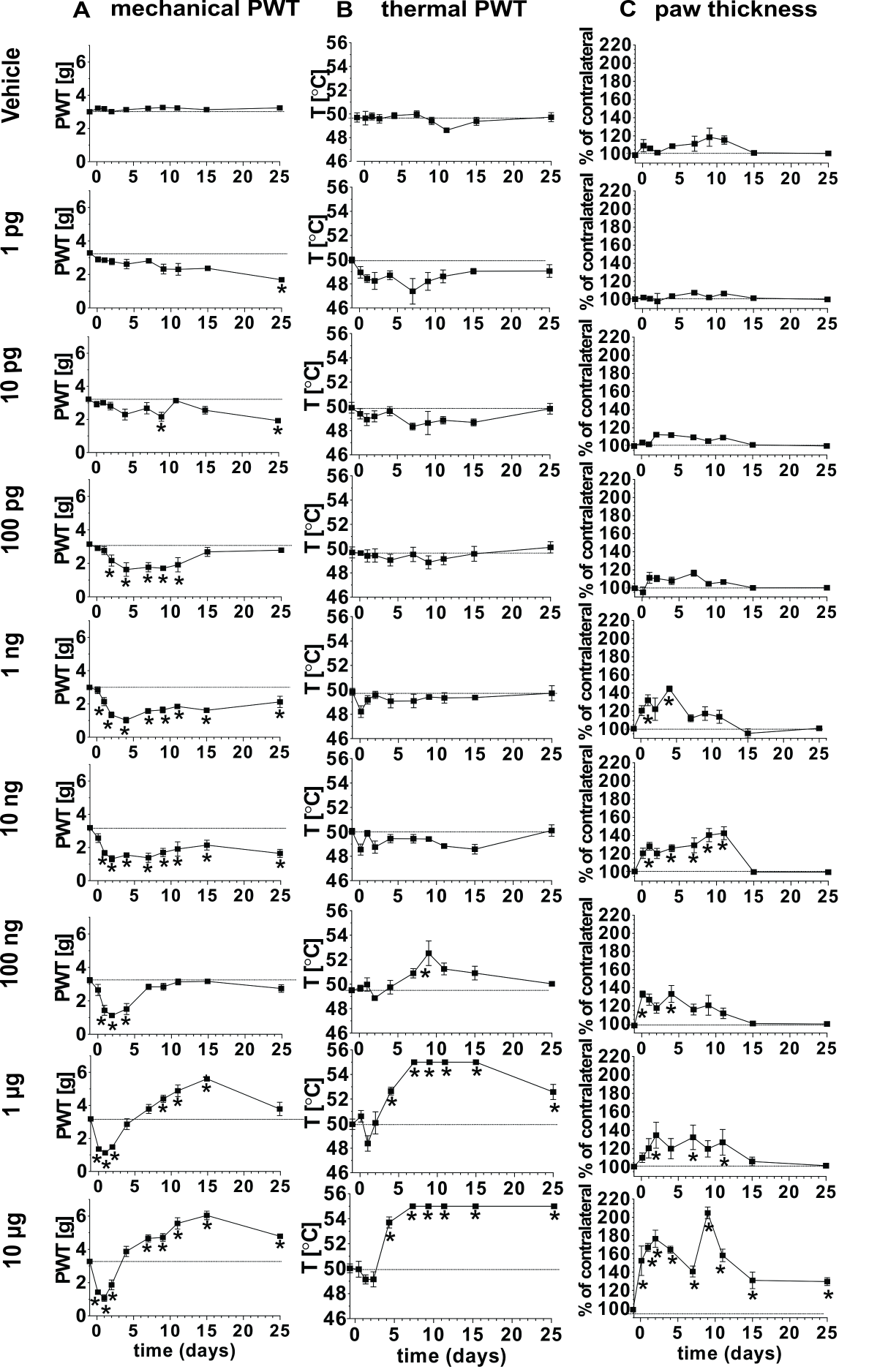

Supplement: Supplementary file 2 [file Image_2.TIF]
