# Supplementary material for: Minocycline Prevents the Development of Mechanical Allodynia in Mouse Models of Vincristine-Induced Peripheral Neuropathy
Source: Front Neurosci. 2019 Jun 27;13:653. doi: 10.3389/fnins.2019.00653 (PMC6610325; doi:10.3389/fnins.2019.00653)
Supplement: Supplementary file 3 [file Table_1.DOCX]

Supplementary Material

**Minocycline prevents the development of mechanical allodynia in a mouse model of vincristine induced peripheral neuropathy**

# Supplementary Data

**Supplementary Figure S1: Local vincristine administration causes bilateral mechanical hyperalgesia with no sex differences**

Single daily injection of vincristine (i.pl; 10 µl solution containing 10 µg were administered using the schedule in **Fig. 1**. Dotted lines indicate baseline values. Mechanical paw withdrawal threshold (PWT) of female and male C57BL6 following vincristine administration of the i.pl. injected (ipsilateral) and not injected (contralateral) hind paw, measured using an electronic von Frey instrument (MouseMet, TopCat Metrology). Statistical significance was determined using two-way ANOVA with Tukey's multiple comparisons test. The values of the ipsilateral hind paw were compared to the values of the contralateral hind paw for each sex separately. No significant differences between the sexes were detected. All data are shown as mean ± SEM; n = 3 for all groups (3 females or 3 males). (**P <  0.05*).

**Supplementary Figure S2: Behavioral characterization of a novel mouse model of vincristine induced peripheral neuropathy.**

Single daily injection of vincristine (i.pl; 10 µl solution containing 1 pg, 10 pg, 100 pg, 1 ng, 10 ng, 100 ng, 1 µg or 10 µg) or vehicle (i.pl; 10 µl 5% glucose) were administered using the schedule in **Fig. 1**. Dotted lines indicate baseline values. **A)** Mechanical paw withdrawal threshold (PWT) following vincristine administration, measured using an electronic von Frey instrument (MouseMet, TopCat Metrology) (black squares) **B)** Thermal paw withdrawal threshold (PWT) following vincristine administration assessed using MouseMet Thermal (TopCat Metrology) (black squares). **C)** Paw thickness 30 min after injection was assessed using a digital Vernier caliper (Kincrome, Vic, Australia) (black squares). ). Statistical significance was determined using two-way ANOVA with Tukey's multiple comparisons test; the experimental groups (vincristine i.pl.) were compared to the vehicle control group (i.pl., 5% glucose). No significant differences between the sexes were detected. All data are shown as mean ± SEM; n = 6 for all groups (3 females and 3 males). (**P <  0.05*).

**Supplementary Table S1**:

Mechanical paw withdrawal threshold (PWT) values following single daily injections of vincristine (i.pl; 10 µl solution containing 10 µg, 1 µg, 100 ng, 1 ng, 100 pg, 10 pg, 1 pg or i.p.; 10 µl/g solution containing 0.5 mg/kg) or vehicle (i.pl; 10 µl 5% glucose or i.p.; 10 µl/g PBS) measured using an electronic von Frey instrument (MouseMet, TopCat Metrology). Data shown as mean and standard error of mean of 6 animals (3 males and 3 females) (**P <  0.05*). VC: vincristine; i.pl.: intraplantar injection; i.p.: intraperitoneal injection.

| **time (days)** | **10 µg VC i.pl.** | | | **1 µg VC i.pl.** | | | **100 ng VC i.pl.** | | | **10 ng VC i.pl.** | | | **1 ng VC i.pl.** | | | **100 pg VC i.pl.** | | |
| --- | --- | --- | --- | --- | --- | --- | --- | --- | --- | --- | --- | --- | --- | --- | --- | --- | --- | --- |
|  | **MEAN** | **SEM** | **N** | **MEAN** | **SEM** | **N** | **MEAN** | **SEM** | **N** | **MEAN** | **SEM** | **N** | **MEAN** | **SEM** | **N** | **MEAN** | **SEM** | **N** |
| **-1** | 3.3 | 0.1 | 6 | 3.2 | 0.1 | 6 | 3.2 | 0.2 | 6 | 3.2 | 0.1 | 6 | 3.0 | 0.1 | 6 | 3.1 | 0.1 | 6 |
| **0** | 1.4* | 0.1 | 6 | 1.4* | 0.1 | 6 | 2.7 | 0.3 | 6 | 2.6 | 0.3 | 6 | 2.8 | 0.2 | 6 | 2.9 | 0.0 | 6 |
| **1** | 1.1* | 0.2 | 6 | 1.1* | 0.1 | 6 | 1.4* | 0.3 | 6 | 1.7* | 0.2 | 6 | 2.1* | 0.2 | 6 | 2.8 | 0.2 | 6 |
| **2** | 1.9* | 0.3 | 6 | 1.5* | 0.1 | 6 | 1.1* | 0.1 | 6 | 1.3* | 0.2 | 6 | 1.3* | 0.2 | 6 | 2.2* | 0.3 | 6 |
| **4** | 3.9 | 0.3 | 6 | 2.9 | 0.3 | 6 | 1.5* | 0.3 | 6 | 1.5* | 0.1 | 6 | 1.0* | 0.2 | 6 | 1.6* | 0.4 | 6 |
| **7** | 4.7* | 0.2 | 6 | 3.8 | 0.3 | 6 | 2.8 | 0.2 | 6 | 1.4* | 0.3 | 6 | 1.6* | 0.2 | 6 | 1.8* | 0.3 | 6 |
| **9** | 4.7* | 0.2 | 6 | 4.4* | 0.2 | 6 | 2.8 | 0.2 | 6 | 1.7* | 0.2 | 6 | 1.6* | 0.2 | 6 | 1.7* | 0.1 | 6 |
| **11** | 5.6* | 0.3 | 6 | 4.9* | 0.4 | 6 | 3.1 | 0.2 | 6 | 1.9* | 0.4 | 6 | 1.9* | 0.1 | 6 | 1.9* | 0.4 | 6 |
| **15** | 6.0* | 0.3 | 6 | 5.6* | 0.1 | 6 | 3.2 | 0.1 | 6 | 2.1* | 0.3 | 6 | 1.6* | 0.1 | 6 | 2.7 | 0.3 | 6 |
| **25.0** | 4.8* | 0.1 | 6 | 3.8 | 0.4 | 6 | 2.7 | 0.2 | 6 | 1.6* | 0.2 | 6 | 2.1* | 0.3 | 6 | 2.8 | 0.1 | 6 |
|  |  |  |  |  |  |  |  |  |  |  |  |  |  |  |  |  |  |  |
| **time (days)** | **10 pg VC i.pl.** | | | **1 pg VC i.pl.** | | | **5% glucose i.pl.** | | | **0.5 mg/kg VC i.p.** | | | **PBS i.p.** | | |  |  |  |
|  | **MEAN** | **SEM** | **N** | **MEAN** | **SEM** | **N** | **MEAN** | **SEM** | **N** | **MEAN** | **SEM** | **N** | **MEAN** | **SEM** | **N** |  |  |  |
| **-1** | 3.2 | 0.1 | 6 | 3.3 | 0.1 | 6 | 3.0 | 0.1 | 6 | 3.4 | 0.1 | 6 | 3.0 | 0.1 | 6 |  |  |  |
| **0** | 2.9 | 0.2 | 6 | 2.9 | 0.2 | 6 | 3.2 | 0.1 | 6 | 3.0 | 0.1 | 6 | 3.2 | 0.1 | 6 |  |  |  |
| **1** | 3.0 | 0.1 | 6 | 2.9 | 0.1 | 6 | 3.2 | 0.1 | 6 | 1.5* | 0.2 | 6 | 3.2 | 0.2 | 6 |  |  |  |
| **2** | 2.8 | 0.2 | 6 | 2.8 | 0.2 | 6 | 3.0 | 0.1 | 6 | 1.3* | 0.1 | 6 | 3.1 | 0.1 | 6 |  |  |  |
| **4** | 2.3 | 0.3 | 6 | 2.6 | 0.3 | 6 | 3.1 | 0.1 | 6 | 1.3* | 0.1 | 6 | 3.1 | 0.1 | 6 |  |  |  |
| **7** | 2.7 | 0.3 | 6 | 2.8 | 0.1 | 6 | 3.2 | 0.1 | 6 | 1.6* | 0.2 | 6 | 3.2 | 0.1 | 6 |  |  |  |
| **9** | 2.2* | 0.3 | 6 | 2.3 | 0.3 | 6 | 3.3 | 0.1 | 6 | 1.9* | 0.2 | 6 | 3.1 | 0.2 | 6 |  |  |  |
| **11** | 3.1 | 0.1 | 6 | 2.3 | 0.3 | 6 | 3.2 | 0.1 | 6 | 1.8* | 0.2 | 6 | 3.1 | 0.1 | 6 |  |  |  |
| **15** | 2.6 | 0.2 | 6 | 2.4 | 0.1 | 6 | 3.1 | 0.1 | 6 | 1.7* | 0.2 | 6 | 3.1 | 0.1 | 6 |  |  |  |
| **25** | 1.9* | 0.1 | 6 | 1.7* | 0.1 | 6 | 3.2 | 0.1 | 6 | 2.4 | 0.2 | 6 | 3.3 | 0.1 | 6 |  |  |  |

**Supplementary Table S2**:

Thermal paw withdrawal threshold (PWT) values following single daily injection of vincristine (i.pl; 10 µl solution containing 10 µg, 1 µg, 100 ng, 1 ng, 100 pg, 10 pg, 1 pg or i.p.; 10 µl/g solution containing 0.5 mg/kg) or vehicle (i.pl; 10 µl 5% glucose or i.p.; 10 µl/g PBS) assessed using MouseMet Thermal (TopCat Metrology). Data shown as mean and standard error of mean of 6 animals (3 males and 3 females) (**P <  0.05*). VC: vincristine; i.pl.: intraplantar injection; i.p.: intraperitoneal injection.

| **time (days)** | **10 µg VC i.pl.** | | | **1 µg VC i.pl.** | | | **100 ng VC i.pl.** | | | **10 ng VC i.pl.** | | | **1 ng VC i.pl.** | | | **100 pg VC i.pl.** | | |
| --- | --- | --- | --- | --- | --- | --- | --- | --- | --- | --- | --- | --- | --- | --- | --- | --- | --- | --- |
|  | **MEAN** | **SEM** | **N** | **MEAN** | **SEM** | **N** | **MEAN** | **SEM** | **N** | **MEAN** | **SEM** | **N** | **MEAN** | **SEM** | **N** | **MEAN** | **SEM** | **N** |
| **-1** | 50.0 | 0.4 | 6 | 49.9 | 0.4 | 6 | 49.5 | 0.2 | 6 | 50.0 | 0.3 | 6 | 49.8 | 0.3 | 6 | 49.7 | 0.5 | 6 |
| **0** | 50.0 | 0.6 | 6 | 50.6 | 0.5 | 6 | 49.7 | 0.3 | 6 | 48.5 | 0.5 | 6 | 48.2 | 0.5 | 6 | 49.6 | 0.1 | 6 |
| **1** | 49.1 | 0.4 | 6 | 48.4 | 0.6 | 6 | 50.0 | 0.5 | 6 | 49.9 | 0.2 | 6 | 49.2 | 0.3 | 6 | 49.4 | 0.5 | 6 |
| **2** | 49.1 | 0.6 | 6 | 50.1 | 0.9 | 6 | 48.9 | 0.2 | 6 | 48.7 | 0.5 | 6 | 49.6 | 0.3 | 6 | 49.4 | 0.5 | 6 |
| **4** | 53.7* | 0.4 | 6 | 52.6* | 0.3 | 6 | 49.7 | 0.6 | 6 | 49.4 | 0.3 | 6 | 49.1 | 0.6 | 6 | 49.1 | 0.5 | 6 |
| **7** | 55.0* | 0.0 | 6 | 55.0* | 0.0 | 6 | 50.9 | 0.4 | 6 | 49.4 | 0.4 | 6 | 49.1 | 0.6 | 6 | 49.5 | 0.6 | 6 |
| **9** | 55.0* | 0.0 | 6 | 55.0* | 0.0 | 6 | 52.5* | 1.0 | 6 | 49.4 | 0.2 | 6 | 49.4 | 0.2 | 6 | 48.9 | 0.5 | 6 |
| **11** | 55.0* | 0.0 | 6 | 55.0* | 0.0 | 6 | 51.2 | 0.5 | 6 | 48.8 | 0.2 | 6 | 49.3 | 0.4 | 6 | 49.2 | 0.5 | 6 |
| **15** | 55.0* | 0.0 | 6 | 55.0* | 0.0 | 6 | 50.9 | 0.6 | 6 | 48.6 | 0.4 | 6 | 49.4 | 0.2 | 6 | 49.6 | 0.6 | 6 |
| **25** | 55.0* | 0.0 | 6 | 52.6* | 0.6 | 6 | 50.0 | 0.1 | 6 | 50.1 | 0.5 | 6 | 49.7 | 0.6 | 6 | 50.1 | 0.5 | 6 |
|  |  |  |  |  |  |  |  |  |  |  |  |  |  |  |  |  |  |  |
| **time (days)** | **10 pg VC i.pl.** | | | **1 pg VC i.pl.** | | | **5% glucose i.pl.** | | | **0.5 mg/kg VC i.p.** | | | **PBS i.p.** | | |  |  |  |
|  | **MEAN** | **SEM** | **N** | **MEAN** | **SEM** | **N** | **MEAN** | **SEM** | **N** | **MEAN** | **SEM** | **N** | **MEAN** | **SEM** | **N** |  |  |  |
| **-1** | 49.9 | 0.4 | 6 | 50.0 | 0.3 | 6 | 49.7 | 0.4 | 6 | 50.4 | 0.2 | 6 | 42.9 | 7.4 | 6 |  |  |  |
| **0** | 49.4 | 0.4 | 6 | 48.9 | 0.5 | 6 | 49.6 | 0.6 | 6 | 50.9 | 0.2 | 6 | 43.0 | 7.4 | 6 |  |  |  |
| **1** | 48.9 | 0.5 | 6 | 48.4 | 0.3 | 6 | 49.8 | 0.3 | 6 | 51.1 | 0.4 | 6 | 42.8 | 7.4 | 6 |  |  |  |
| **2** | 49.2 | 0.5 | 6 | 48.2 | 0.7 | 6 | 49.6 | 0.4 | 6 | 50.4 | 0.4 | 6 | 42.7 | 7.3 | 6 |  |  |  |
| **4** | 49.6 | 0.4 | 6 | 48.7 | 0.4 | 6 | 49.9 | 0.3 | 6 | 50.4 | 0.5 | 6 | 42.9 | 7.4 | 6 |  |  |  |
| **7** | 48.3 | 0.3 | 6 | 47.4 | 1.1 | 6 | 50.0 | 0.3 | 6 | 49.4 | 0.4 | 6 | 42.8 | 7.4 | 6 |  |  |  |
| **9** | 48.6 | 1.0 | 6 | 48.2 | 0.7 | 6 | 49.4 | 0.3 | 6 | 49.0 | 0.4 | 6 | 42.3 | 7.3 | 6 |  |  |  |
| **11** | 48.9 | 0.3 | 6 | 48.6 | 0.5 | 6 | 48.6 | 0.2 | 6 | 49.2 | 0.4 | 6 | 41.9 | 7.2 | 6 |  |  |  |
| **15** | 48.7 | 0.3 | 6 | 49.0 | 0.2 | 6 | 49.4 | 0.3 | 6 | 49.6 | 0.3 | 6 | 42.3 | 7.3 | 6 |  |  |  |
| **25** | 49.8 | 0.4 | 6 | 49.1 | 0.5 | 6 | 49.7 | 0.4 | 6 | 49.9 | 0.4 | 6 | 42.6 | 7.3 | 6 |  |  |  |

**Supplementary Table S3**:

Paw thickness values 30 min after single daily injection of vincristine (i.pl; 10 µl solution containing 10 µg, 1 µg, 100 ng, 1 ng, 100 pg, 10 pg, 1 pg or i.p.; 10 µl/g solution containing 0.5mg/kg) or vehicle (i.pl; 10 µl 5% glucose or i.p.; 10 µl/g PBS) was assessed using a digital Vernier caliper (Kincrome, Vic, Australia). Data shown as mean and standard error of mean in percent of the contralateral hind paw of 6 animals (3 males and 3 females).Values expressed as percent of the contralateral hind paw (**P <  0.05*). VC: vincristine; i.pl.: intraplantar injection; i.p.: intraperitoneal injection.

| **time (days)** | **10 µg VC i.pl.** | | | **1 µg VC i.pl.** | | | **100 ng VC i.pl.** | | | **10 ng VC i.pl.** | | | **1 ng VC i.pl.** | | | **100 pg VC i.pl.** | | |
| --- | --- | --- | --- | --- | --- | --- | --- | --- | --- | --- | --- | --- | --- | --- | --- | --- | --- | --- |
|  | **MEAN** | **SEM** | **N** | **MEAN** | **SEM** | **N** | **MEAN** | **SEM** | **N** | **MEAN** | **SEM** | **N** | **MEAN** | **SEM** | **N** | **MEAN** | **SEM** | **N** |
| **-1** | 98.8 | 0.9 | 6 | 100.6 | 1.1 | 6 | 98.3 | 1.0 | 6 | 100.6 | 0.3 | 6 | 100.6 | 0.4 | 6 | 99.6 | 0.2 | 6 |
| **0** | 152.4* | 16.1 | 6 | 110.3 | 5.2 | 6 | 126.8 | 6.2 | 6 | 120.6 | 5.6 | 6 | 120.6 | 5.5 | 6 | 95.1 | 5.9 | 6 |
| **1** | 167.0* | 4.4 | 6 | 120.5 | 10.5 | 6 | 139.9* | 3.5 | 6 | 128.3* | 4.2 | 6 | 132.0 | 6.0 | 6 | 111.2 | 6.0 | 6 |
| **2** | 176.5* | 9.4 | 6 | 134.7* | 14.1 | 6 | 117.6 | 5.6 | 6 | 120.4 | 5.5 | 6 | 122.2 | 12.4 | 6 | 110.4 | 3.5 | 6 |
| **4** | 164.5* | 3.8 | 6 | 120.1 | 11.0 | 6 | 143.2* | 9.3 | 6 | 126.2* | 3.8 | 6 | 144.7* | 3.1 | 6 | 107.9 | 4.1 | 6 |
| **7** | 140.6* | 6.0 | 6 | 132.4* | 13.3 | 6 | 115.9 | 6.0 | 6 | 129.4* | 8.2 | 6 | 112.1 | 3.9 | 6 | 116.4 | 3.4 | 6 |
| **9** | 204.7* | 6.4 | 6 | 119.9 | 8.9 | 6 | 120.6 | 11.0 | 6 | 140.6* | 7.3 | 6 | 117.3 | 7.5 | 6 | 104.6 | 1.1 | 6 |
| **11** | 158.3* | 6.9 | 6 | 127.0* | 13.9 | 6 | 111.7 | 5.7 | 6 | 142.6* | 7.1 | 6 | 113.7 | 7.2 | 6 | 106.6 | 2.0 | 6 |
| **15** | 130.9* | 9.1 | 6 | 106.2 | 4.7 | 6 | 100.5 | 0.6 | 6 | 100.0 | 0.7 | 6 | 95.4 | 5.1 | 6 | 100.1 | 0.8 | 6 |
| **25** | 129.6* | 4.3 | 6 | 101.7 | 2.4 | 6 | 100.0 | 0.8 | 6 | 99.8 | 0.3 | 6 | 101.0 | 0.5 | 6 | 100.2 | 1.7 | 6 |
|  |  |  |  |  |  |  |  |  |  |  |  |  |  |  |  |  |  |  |
| **time (days)** | **10 pg VC i.pl.** | | | **1 pg VC i.pl.** | | | **5% glucose i.pl.** | | | **0.5 mg/kg VC i.p.** | | | **PBS i.p.** | | |  |  |  |
|  | **MEAN** | **SEM** | **N** | **MEAN** | **SEM** | **N** | **MEAN** | **SEM** | **N** | **MEAN** | **SEM** | **N** | **MEAN** | **SEM** | **N** |  |  |  |
| **-1** | 99.8 | 0.2 | 6 | 100.3 | 0.5 | 6 | 98.8 | 1.7 | 6 | 99.7 | 0.8 | 6 | 99.1 | 0.9 | 6 |  |  |  |
| **0** | 103.8 | 1.9 | 6 | 102.2 | 2.5 | 6 | 109.2 | 6.8 | 6 | 100.3 | 0.5 | 6 | 100.4 | 0.4 | 6 |  |  |  |
| **1** | 101.8 | 1.6 | 6 | 100.8 | 0.9 | 6 | 106.1 | 2.6 | 6 | 100.7 | 0.5 | 6 | 99.8 | 0.6 | 6 |  |  |  |
| **2** | 112.3 | 2.4 | 6 | 97.9 | 8.8 | 6 | 101.5 | 0.9 | 6 | 99.7 | 0.6 | 6 | 99.6 | 0.7 | 6 |  |  |  |
| **4** | 111.9 | 2.1 | 6 | 103.6 | 1.7 | 6 | 108.7 | 2.6 | 6 | 99.6 | 0.4 | 6 | 99.4 | 0.4 | 6 |  |  |  |
| **7** | 109.4 | 1.8 | 6 | 107.5 | 0.7 | 6 | 111.3 | 8.3 | 6 | 99.5 | 0.3 | 6 | 100.6 | 1.2 | 6 |  |  |  |
| **9** | 105.3 | 2.1 | 6 | 102.2 | 1.5 | 6 | 118.5 | 10.0 | 6 | 99.9 | 0.4 | 6 | 100.5 | 1.0 | 6 |  |  |  |
| **11** | 109.2 | 1.3 | 6 | 106.5 | 1.3 | 6 | 115.4 | 4.6 | 6 | 100.2 | 0.3 | 6 | 100.4 | 0.9 | 6 |  |  |  |
| **15** | 101.1 | 0.7 | 6 | 101.4 | 1.2 | 6 | 101.2 | 1.0 | 6 | 99.9 | 0.4 | 6 | 100.4 | 0.6 | 6 |  |  |  |
| **25** | 99.9 | 0.2 | 6 | 100.1 | 0.4 | 6 | 100.7 | 0.6 | 6 | 100.2 | 0.4 | 6 | 99.8 | 0.5 | 6 |  |  |  |

**Supplementary Table S4**:

Ipsilateral paw print area expressed in percent of the contralateral paw following daily injections of vincristine (i.pl; 10 µl solution containing 10 µg, 1 µg, 100 ng or i.p.; 10 µl/g solution containing 0.5 mg/kg) or vehicle (i.pl; 10 µl 5% glucose or i.p.; 10 µl/g PBS) was assessed using the CatwalkXT platform (Noldus Information Technology, Netherlands). Print area is the average of paw contact area with the glass platform (in cm^2^). Data shown as mean and standard error in percent of the contralateral hind paw of 6 animals (3 males and 3 females). Values expressed as percent of the contralateral hind paw. (**P <  0.05*)

| **time (days)** | **10 µg VC i.pl.** | | | **1 µg VC i.pl.** | | | **100 ng VC i.pl.** | | | **5% glucose i.pl.** | | | **0.5 mg/kg VC i.p.** | | | **PBS i.p.** | | |
| --- | --- | --- | --- | --- | --- | --- | --- | --- | --- | --- | --- | --- | --- | --- | --- | --- | --- | --- |
|  | **MEAN** | **SEM** | **N** | **MEAN** | **SEM** | **N** | **MEAN** | **SEM** | **N** | **MEAN** | **SEM** | **N** | **MEAN** | **SEM** | **N** | **MEAN** | **SEM** | **N** |
| **-1** | 102.8 | 6.5 | 6 | 110.2 | 6.6 | 6 | 121.6 | 12.3 | 6 | 110.2 | 2.9 | 6 | 112.2 | 11.3 | 6 | 112.2 | 11.3 | 6 |
| **0** | 95.5 | 3.6 | 6 | 101.4 | 4.4 | 6 | 105.1 | 4.8 | 6 | 96.7 | 8.0 | 6 | 98.1 | 6.7 | 6 | 105.7 | 6.0 | 6 |
| **1** | 84.5 | 4.6 | 6 | 96.6 | 7.3 | 6 | 92.6 | 6.5 | 6 | 86.3 | 5.5 | 6 | 99.2 | 5.9 | 6 | 90.6 | 4.4 | 6 |
| **2** | 95.5 | 3.6 | 6 | 96.3 | 6.4 | 6 | 95.5 | 6.7 | 6 | 99.0 | 7.8 | 6 | 101.6 | 6.9 | 6 | 94.8 | 8.9 | 6 |
| **4** | 18.5* | 7.2 | 6 | 92.5 | 9.5 | 6 | 96.9 | 5.0 | 6 | 102.7 | 12.6 | 6 | 99.1 | 11.8 | 6 | 95.7 | 5.6 | 6 |
| **7** | 15.8* | 4.7 | 6 | 88.2* | 8.0 | 6 | 105.3 | 6.3 | 6 | 111.4 | 5.5 | 6 | 101.9 | 5.9 | 6 | 112.0 | 7.0 | 6 |
| **9** | 13.3* | 3.4 | 6 | 88.8* | 9.5 | 6 | 97.6 | 4.0 | 6 | 103.5 | 5.5 | 6 | 105.8 | 6.0 | 6 | 104.2 | 5.4 | 6 |
| **11** | 1.2* | 1.2 | 6 | 67.3* | 8.9 | 6 | 98.7 | 7.7 | 6 | 103.2 | 4.1 | 6 | 104.7 | 7.9 | 6 | 100.5 | 3.7 | 6 |
| **15** | 1.6* | 1.6 | 6 | 106.8 | 3.5 | 6 | 92.6 | 7.9 | 6 | 100.9 | 5.4 | 6 | 93.9 | 7.1 | 6 | 105.5 | 1.8 | 6 |
| **25** | 19.4* | 9.0 | 6 | 78.7* | 3.4 | 6 | 107.1 | 13.2 | 6 | 99.2 | 9.7 | 6 | 83.3 | 10.0 | 6 | 90.7 | 10.1 | 6 |

VC: vincristine; i.pl.: intraplantar injection; i.p.: intraperitoneal injection.

**Supplementary Table S5**: Evaluation of local or systemic vincristine (i.pl., 10 μg, 100 ng or i.p., 0.5 mg/kg) or vehicle (i.pl., 5% glucose or i.p. PBS) histopathological changes in the injected hind paws by H&E staining. **–**: absent, **+**: minor, **++**: mild, **+++**: moderate, **++++**: extensive. PBS: phosphate buffered saline; i.pl.: intraplantar injection; i.p.: intraperitoneal injection.

| Treatment | | **Vehicle** | | | | **Vincristine** | | | | | |
| --- | --- | --- | --- | --- | --- | --- | --- | --- | --- | --- | --- |
|  |  | 5% Glucose i.pl. | | PBS i.p. | | 100 ng i.pl. | | 10 µg i.pl. | | 0.5 mg/kg i.p. | |
| Time point | | **24h** | **7 D** | **24h** | **7 D** | **24h** | **7 D** | **24h** | **7 D** | **24h** | **7 D** |
| Morphological change | Muscle edema | ₋ | ₊ | ₊ | ₊ | ₊ | ₊₊₊ | ₊₊ | ₊₊₊ | ₊ | ₊ |
|  | Nerve edema | ₋ | ₋ | ₋ | ₋ | ₋ | ₋ | ₋ | ₊ | ₋ | ₋ |
|  | Nerve vacuolization | ₋ | ₋ | ₋ | ₋ | ₋ | ₊ | ₊ | ₊ | ₋ | ₋ |
|  | Dermal edema | ₋ | ₋ | ₋ | ₋ | ₊ | ₊₊₊ | ₊₊ | ₊₊₊ | ₊ | ₊ |
|  | Dermal necrosis | ₋ | ₋ | ₋ | ₋ | ₋ | ₋ | ₋ | ₊₊₊₊ | ₋ | ₋ |
|  | Dermal infiltrate | ₊ | ₊ | ₋ | ₋ | ₊ | ₊₊ | ₊ | ₊₊₊ | ₋ | ₋ |
|  | Fibroblast reactivity | ₋ | ₊ | ₋ | ₋ | ₋ | ₊ | ₋ | ₊ | ₋ | ₋ |
|  | Vascular degeneration | ₋ | ₋ | ₋ | ₋ | ₋ | ₋ | ₊ | ₊₊₊₊ | ₋ | ₋ |
